# Commentary: Acrylamide formation in air-fried versus deep and oven-fried potatoes

**DOI:** 10.3389/fnut.2024.1397302

**Published:** 2024-06-12

**Authors:** Ceyhan Ceran Serdar

**Affiliations:** Department of Medical Biology and Genetics, School of Medicine, Ankara Medipol University, Ankara, Türkiye

**Keywords:** acrylamide, LoD, LoQ, potato chip, air frying, oven frying, deep frying of potatoes

The recent surge in popularity of air fryers, attributed to their ease of use and the widespread belief in their health benefits, has led to increased interest in the health implications of air-fried foods. A recent study by Navruz-Varlı and Mortaş (1) titled “*Acrylamide formation in air-fried versus deep and oven-fried potatoes*”, which investigated the health aspects of air-fried products, was particularly engaging for scientists aiming to understand the potential benefits and risks associated with this cooking method.

I would like to thank the authors for their contributions to your journal. It caught my attention that this article is being referenced by dietitians on social media. Recognizing the public's heightened interest in research with practical applications and aiming to ensure the responsible translation of the study's conclusions into both personal practice and potential dissemination to the broader community, avoiding any unintended implications, I critically evaluated the study from the perspective of a diagnostic kit developer. As a result, I would like to raise some points for your consideration as well as your readers' consideration.

1. In the article, the limit of detection (LoD) and limit of quantification (LoQ) values for acrylamide measurement were provided as 4.84 ng/g (μg/kg) and 18.20 ng/g (μg/kg), respectively (1). All the mean acrylamide levels presented in both the tables and the graphs are significantly lower than the stated LoQ values of 18.20 ng/g (μg/kg) (1). By definition, “limit of quantitation” refers to the lowest limit of an analyte that can be precisely quantified by the method of interest. Analyte levels falling between the LoD and LoQ can only be claimed to be present in the sample, yet it is not valid to state a precise quantity for them. This is because their levels are too low to be precisely determined by the method of interest. To illustrate, a sample measured slightly above the LoD in the first measurement might fall below the LoQ when reanalyzed. Consequently, acrylamide levels between 4.84 ng/g (μg/kg) and 18.20 ng/g (μg/kg) cannot be distinguished using the analytical method employed in this study. Despite this limitation, all mean acrylamide values provided for the differently treated potato samples fall below the LoQ limit of 18.20 ng/g (μg/kg), indicating that they cannot be differentiated from each other. Notably, the article emphasizes that the acrylamide level in the samples subjected to soaking in the “Deep frying” group is statistically significantly lower than the samples subjected to washing. However, the acrylamide level of the former (1.18 ± 0.18 μg/kg) falls even below the LoD, signifying non-detectable concentrations.
2. The authors refer to a previous study by Rufián-Henares and Morales for the determination of LoQ and LoD. In this study, spiked potato homogenates, referred to as “reference material,” were used with increasing concentrations of acrylamide to determine the analytical performance values (LoD and LoQ) of the acrylamide-measurement assays (2). It is noteworthy that the starting potato chip sample, referred to as “reference material” in the referenced study, was not a true “blank” sample with “0 μg/kg acrylamide”; in fact, it already contained 575 ± 37 μg/kg acrylamide to start with, as determined by an alternative method (2). As LoD and LoQ cannot technically be determined in the absence of a true blank sample, it is crucial that the exact methodology for the determination of LoD and LoQ in the current study be elaborately explained.
3. The acrylamide levels reported in the study seem excessively low and inconsistent with the previous literature. They are approximately 10 times lower than those reported by Giovanelli et al. (3) and Sansano et al. (4). Acrylamide levels in sunflower oil were previously reported to be between 890 and 1,200 μg/kg (5). This suggests that even slight cross-contamination from the sunflower oil used to cook the potatoes could easily be the sole reason for the acrylamide levels measured between 1.18 μg/kg and 16.69 μg/kg in the current study. On the other hand, the FDA report (6) states that chips/French fries are not devoid of acrylamide; in fact, acrylamide levels in chips/French fries were reported to be considerably high, ranging from 59 to 5200 μg/kg (6). The original literature that the authors of the current article refer to for LoD and LoQ determination also mentions that the acrylamide level in the reference potato chips sample was 575 ± 37 μg/kg, significantly higher than the acrylamide levels reported in the current article for potato samples (2). This significant discrepancy between the previous literature and the results published in the current study suggests that the acrylamide levels reported in the current study are either improperly measured or inaccurate, potentially leading to misleading conclusions.
4. In the limitations section, the authors state that “each processing procedure was applied in four different groups, totally 24 samples”. However, the acrylamide results corresponding to the six groups (three different frying conditions, each having two different pre-processing options) were presented throughout the manuscript. Considering a total of 24 samples, the sample size of each tested condition will vary depending on the total number of groups (the sample size per group would be six for a study involving four different groups, while the sample size per group would be four for a study involving six different groups). It would be helpful to clarify the sample size per group, as there seems to be a slight discrepancy between the stated total number of samples (24) and the number of groups presented with acrylamide results (6). This clarification would ensure that readers have all the necessary information to fully interpret the results.

This study delves into a topic that has garnered significant public interest, making it a potential reference for diverse audiences with varying levels of scientific expertise. Recognizing its potential reach beyond the academic community, it is crucial to address the previously discussed inconsistencies to ensure clarity and prevent misinterpretations. By meticulously addressing these discrepancies, this publication can achieve its intended impact and facilitate accurate understanding among a broad readership. This rigorous approach safeguards the integrity of the research and its dissemination throughout society.

## Author contributions

CCS: Writing – original draft, Writing – review & editing.
